# Modified Completion Test (MCT) in Psychological Diagnostics of Patients with Paranoid Schizophrenia — Stage of Retelling the Story

**DOI:** 10.1192/j.eurpsy.2022.1995

**Published:** 2022-09-01

**Authors:** N. Burlakova

**Affiliations:** Lomonosov Moscow State University, Faculty of Psychology, Department Of Neuro- And Pathopsychology, Moscow, Russian Federation

**Keywords:** schizophrénia, cognitive functions, thought disorder, cognitive assessment

## Abstract

**Introduction:**

The modified completion test (MCT) based on the stories by H. Ebbinghaus enables to assess cognitive functions in situation close to a real-life task with an affective load (Burlakova,2016,2020). MCT includes the following stages: 1) filling the gaps in the story; 2) reading and retelling; 3) making up a continuation and a title; 4) retelling the story and its continuation after 30–40 minutes.

**Objectives:**

The objective was to research diagnostical potential of the second stage of MCT for patients suffering from paranoid schizophrenia with hallucinatory syndrome.

**Methods:**

The study included 42 patients (28 female, 14 male) with schizophrenia (disease onset at least 5–7 years ago), aged from 19 to 51 (average age 35±8), receiving treatment. Control group consisted of 44 people (average age 37±6), never sought psychiatric help, never diagnosed with any mental disorders. Groups were organized to be equal in gender proportions, age, and educational level.

**Results:**

In comparison to the control group, the psychiatric patients demonstrated: 1) lower connectedness in narration, lower ability to reproduce main elements of the plot; 2) unusual logic in introduction of new details, extensiveness of such details; 3) lower integrity of mnestic functions, lower ability to maintain concentration. The clinical group: 1) imposed on the text principally different logic, subjectively significant, yet far from the original context; 2) suddenly introduced of new ideas; 3) had confabulations; 4) were altiloquent.

**Conclusions:**

The stage of retelling enables to assess semantic memory, regulatory functions, connectedness of the narration, cogitation and to examine cognitive functions in the context of patient’s personality.

**Disclosure:**

No significant relationships.

